# The Central Nervous System as Target of *Bacillus anthracis* Toxin Independent Virulence in Rabbits and Guinea Pigs

**DOI:** 10.1371/journal.pone.0112319

**Published:** 2014-11-06

**Authors:** Haim Levy, Itai Glinert, Shay Weiss, Elad Bar-David, Assa Sittner, Josef Schlomovitz, Zeev Altboum, David Kobiler

**Affiliations:** Department of Infectious Diseases, Israel Institute for Biological Research, Ness Ziona, Israel; Rutgers University, United States of America

## Abstract

Infection of the central nervous system is considered a complication of Anthrax and was reported in humans and non-human primates. Previously we have reported that *Bacillus anthracis* possesses a toxin-independent virulent trait that, like the toxins, is regulated by the major virulence regulator, AtxA, in the presence of pXO2. This toxin-independent lethal trait is exhibited in rabbits and Guinea pigs following significant bacteremia and organ dissemination. Various findings, including meningitis seen in humans and primates, suggested that the CNS is a possible target for this AtxA-mediated activity. In order to penetrate into the brain tissue, the bacteria have to overcome the barriers isolating the CNS from the blood stream. Taking a systematic genetic approach, we compared intracranial (IC) inoculation and IV/SC inoculation for the outcome of the infection in rabbits/GP, respectively. The outstanding difference between the two models is exhibited by the encapsulated strain VollumΔpXO1, which is lethal when injected IC, but asymptomatic when inoculated IV/SC. The findings demonstrate that there is an apparent bottleneck in the ability of mutants to penetrate into the brain. Any mutant carrying either pXO1 or pXO2 will kill the host upon IC injection, but only those carrying AtxA either on pXO1 or in the chromosome in the background of pXO2 can penetrate into the brain following peripheral inoculation. The findings were corroborated by histological examination by H&E staining and immunofluorescence of rabbits' brains following IV and IC inoculations. These findings may have major implications on future research both on *B. anthracis* pathogenicity and on vaccine development.

## Introduction

Anthrax is a zoonotic disease caused by the gram-positive spore-forming bacterium *Bacillus anthracis*. In humans, four types of anthrax have been recorded according to the route of infection: cutaneous, gastrointestinal, inhalation and intravenous [1]. The accepted paradigm states that anthrax is both an invasive and toxinogenic disease and that the toxins play major roles in pathogenicity. In previous studies, we tested this assumption by a systematic genetic study deleting the toxin genes in a fully virulent Vollum strain, testing the virulence of the mutant strains in guinea pigs (GP) and rabbits. In both models, full virulence requires both toxins, Lethal toxin (LT) and Edema toxin (ET), but either one is sufficient for virulence (with LD_50_ increased to about 10 to 100 folds). In both models, deletion of the all three toxin components, *pag, lef* and *cya* genes (protective antigen, lethal and edema factors, respectively), results in moderate attenuation, maintaining significant virulence of these mutants, as estimated by death of infected animals [2]. The finding that virulence is maintained after fully deleting toxin components suggests that *B*. *anthracis* possesses an additional virulence mechanism, independent of the three main toxin components. Previous results [3] show that this mechanism is pXO1 dependent, as the encapsulated pXO1^−^pXO2^+^mutant shows complete attenuation. In addition, this virulence was shown to depend on AtxA activity in the presence of pXO2, as deletion of either *atxA* or pXO2 results in complete loss of virulence in the absence of toxins. Furthermore, insertion of AtxA into the non-virulent VollumΔpXO1 results in recovery of the virulent trait, demonstrating that AtxA is the only gene from pXO1 required for the exhibition of the toxin independent virulence. The ability of toxin-deficient mutants to effectively kill experimental hosts, coupled with abundant data regarding the function of these toxins on various systems, and especially the immune system, leads to the assumption that the main contribution of the toxins to anthrax infection is in the early stages [2], [4]. This contribution includes the initial confrontation between spores/bacteria and innate host defenses and their neutralization/evasion by the pathogen. On the other hand, during the septic stage of the disease, either the toxins or the capsule (both regulated by AtxA) can afford protection to the bacteria against the immune system, facilitating hematogenous spread and persistence of the bacilli in different host tissues/organs or their vasculature. Subsequently, AtxA activated genes of unknown genomic location [5] would exert this toxin-independent virulent trait.

A possible target for this AtxA-mediated activity is the brain, an organ sensitive to bacterial infection. In order to penetrate into the brain tissue, the bacteria have to overcome the barriers isolating the CNS from the blood stream, like the blood-brain barrier (BBB), the blood-cerebrospinal fluid barrier (BCSFB) or the blood-arachnoid barrier (BAB) [6]. BBB is established by specialized endothelial cells of CNS micro-vessels, whereas the BCSFB is formed by the epithelial cells of the choroid plexus. The BAB is an arachnoid barrier cell layer between the CSF in the subarachnoid space and the blood circulation in the dura. These cell layers constitute physical barriers sealed by a complex network of tight junctions between adjacent cells.

The hypothesis that the brain is the target for *B. anthracis* is supported by the finding that in all the PA-immunized GP that succumbed to intranasal (IN) challenge, high bacterial counts were found in the brain (this paper). Furthermore, similar results were obtained in brains of rabbits that were inoculated intravenously (IV) with virulent mutants of the Vollum strain [3]. In addition, high rates of meningitis have been reported in humans [7]–[9] and non-human primates ("cardinal's cap"), with occasional local extension of infection into the neuropil [10]. Herein, we report an attempt to test the hypothesis that brain invasion is a toxin-independent phenomenon with similar fatal consequences on rabbits and GP by using of intracerebral/intracranial (IC) inoculation and comparing the outcome to the pattern exhibited following IV inoculation.

A lethal outcome due to an infection of the brain could result from several distinct mechanisms, including direct/active invasion of the meninges and/or the brain parenchyma, or indirect, more passive mechanisms such as vascular adhesion and multiplication causing blood vessel occlusion or induction of exaggerated immune responses causing lethality. Using IC inoculation of various mutants, we were able to demonstrate that there is an apparent bottleneck in the ability of mutants to penetrate into the brain. Any mutant carrying either pXO1 or pXO2 will kill the host upon IC injection, but only those carrying AtxA can penetrate into the brain following bacteremia.

## Results

**Figure 1:**
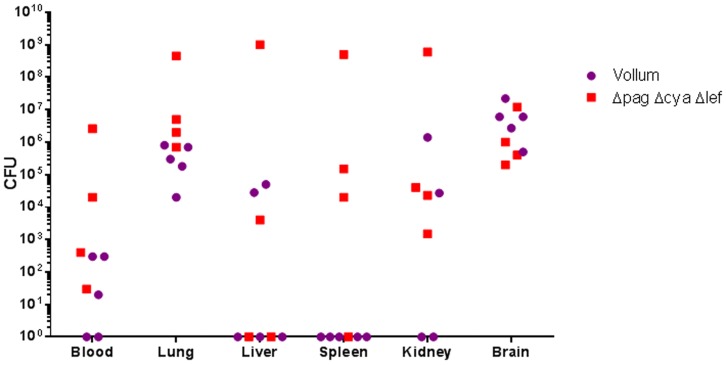
Organ dissemination of the wild type Vollum strain and the toxins-less mutant in PA immunized Guinea pigs. Guinea pigs were immunized with alum absorbed PA at time 0 and 4 weeks and challenged at week 6 by intranasal instillation of spores (10^7^ CFU – 500 LD_50_ of the WT strain and 10^8^ CFU – 2 LD_50_ of the toxins deficient mutant strain). Serum αPA titers were higher than 100,000. Bacterial burden were determined post mortem at various tissues of individual animals.

Immunization of rabbits and GP with a PA-based vaccine was previously demonstrated to protect against challenge with lethal doses of *B. anthracis* spores [11], [12]. However, inoculation of immunized GP with high doses of *B. anthracis* spores by intranasal instillation results in the death of the challenged animals. A dose of 10^7^ CFU Vollum-wt spores killed the infected GP (5/5), whereas an inoculum of 10^8^ VollumΔ*pag*Δ*lef*Δ*cya* spores resulted in a lethality rate of 80% (4/5). Upon death, the bacterial load in blood and organs (lungs, liver, spleen, kidneys and brain) was determined (**Fig. 1**). As can be seen all animals show high variation in the bacterial distribution in the different tissues. However, all dead animals show significant accumulation of bacteria in the lungs and brain, indicating a possible correlation between the death of the GP and the bacterial load in the brain (presence in the lungs may be the consequence of IN inoculation).

**Figure 2:**
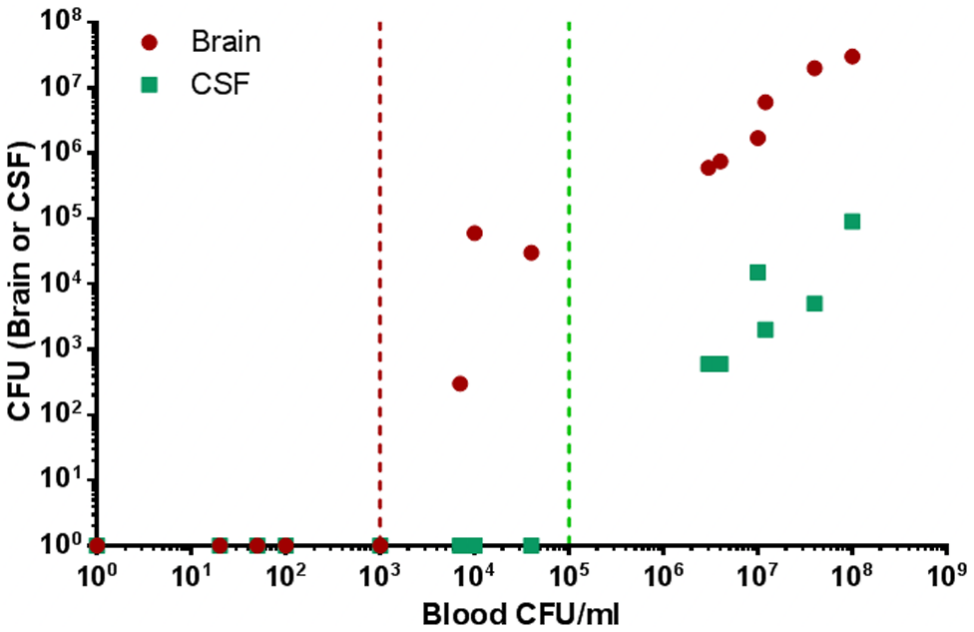
Bacterial load in the brain or CSF as a function of blood bacteremia in rabbits following intranasal instillation of Vollum spores. Rabbits were infected IN with 10^7^ CFU of Vollum spores (500 LD_50_). 32 h post infection blood, CSF and brain total bacterial load were determined. The brain (total CFU) and CSF (CFU/ml) of individual animal is plotted in correlation to the blood bacteremia. The maximal blood bacteremia that does not result in the appearance of bacteria in brain (red) and in CSF (green) is marked with a vertical line.

Previously we developed a rabbit model to study the correlation between bacteremia and the severity of anthrax and the efficacy of therapeutic treatments on the disease [13]. In order to demonstrate that blood-borne *B. anthracis* bacteria can penetrate the brain tissue (by breaching the brain barriers), we monitored the correlation between the bacterial concentration in blood, brain and CSF following IN instillation of Vollum-wt spores (10^7^ CFU) in naive rabbits (**Fig. 2**). At low levels of bacteremia, *B. anthracis* cells could not be detected in the brain. The appearance of bacteria in the brain is followed by a quick and abrupt increment in bacterial concentration, surpassing the contribution of the blood bacteremia in the brain vessels, which constitute 0.1–1% of the brain volume. Furthermore, high levels of bacteria in the brain are accompanied by the presence of bacteria in the CSF (**Fig. 2**). These findings, that were previously reported for other neuro-invasive bacteria [14], indicate that bacterial invasion into the brain tissue is a step in the development of anthrax.

To bypass the step of CNS invasion and evaluate the possibility that the brain is a target for *B. anthracis* infection we studied the outcome of intracranial (IC) inoculation of encapsulated vegetative bacteria in GP and rabbits. Using the IC inoculation model, we evaluated the involvement of pXO1 and the toxins in the bacterial capacity to cause death in inoculated GP. Vegetative bacilli were cultured in inductive conditions (inducing capsule and toxin expression) and were injected IC to GP to test their ability to cause a lethal disease. The results, shown in **Table 1**, demonstrate that injection of mutants carrying either pXO1 or pXO2 has a lethal effect on GP, whereas deletion of both plasmids abolished virulence entirely. The lethal effect of the VollumΔpXO2 mutant is toxin dependent, as the additional deletion of the *pag, lef* and *cya* genes abolishes this effect.

**Table 1:** Susceptibility of GP and rabbits to Vollum strains in the IC injection model.

**Guinea Pigs**				
**Strain**	**Description**	**Inoculum (CFU)**	**Dead/infected**	**TTD (days)**
Vollum	pXO1^+^pXO2^+^	10^5^	4/4	1
Vollum ΔpXO1	pXO1^−^pXO2^+^	10^5^–10^3^	4/4	1
Vollum ΔpXO2	pXO1^+^pXO2^−^	10^5^	4/4	1
VollumΔ*pag*Δ*lef*Δ*cya*	Complete deletion of the *pag, lef* and *cya* genes	10^5^–10^3^	4/4	1
Vollum ΔpXO2 Δ*pag*Δ*lef*Δ*cya*	Complete deletion of pXO2 and the *pag, lef* and *cya* genes	10^5^	0/4	>14
Vollum ΔpXO1 ΔpXO2	pXO1^−^pXO2^−^	10^5^	0/4	>14
**Rabbits**				
**Strain**	**Characterization**	**Inoculum (CFU)**	**Dead/infected**	**TTD (days)**
Vollum	pXO1^+^pXO2^+^	10^5^	4/4	1
Vollum ΔpXO1	pXO1^−^pXO2^+^	10^5^	4/4	1
Vollum ΔpXO2	pXO1^+^pXO2^−^	10^5^	5/6	1–6
VollumΔ*pag*Δ*lef*Δ*cya*	Complete deletion of the *pag, lef* and *cya* genes	10^5^	4/4	1–2
Vollum ΔpXO2 Δ*pag*Δ*lef*Δ*cya*	Complete deletion of pXO2 and the *pag, lef* and *cya* genes	10^5^	0/4	>14
Vollum ΔpXO1 ΔpXO2	pXO1^−^pXO2^−^	10^5^	0/4	>14

In a previous study [3], we tested the same mutants in a GP SC inoculation model, and we found a strict dependence of the virulence on AtxA activity (by comparing the ΔpXO1 and ΔpXO1 ba2805::atxA strains), a dependence not seen in the IC model. The outstanding difference between the two models is exhibited by the encapsulated strain VollumΔpXO1, which is lethal when injected IC, but asymptomatic when inoculated SC. This discrepancy may indicate that this mutant lacks the ability to cause a lethal brain infection from the circulation, possibly due to an inability to cross the brain barrier. Consequently, we can conclude that this "neuro-invasiveness" is an AtxA-regulated trait. Indeed, all IC virulent mutants carrying the *atxA* gene will kill the GP when injected SC.

**Figure 3:**
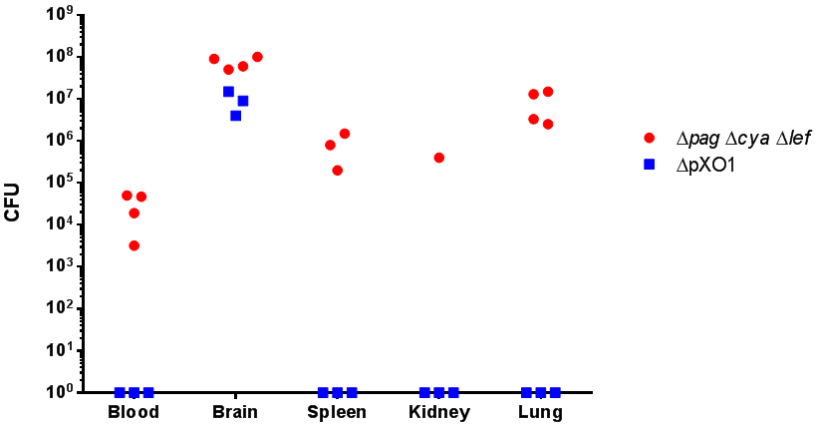
Bacterial dissemination to different organs following IC infection of Guinea pigs. Guinea pigs were inoculated IC with capsular bacteria of the different Vollum strain mutants (Δ*pag*Δ*lef*Δ*cya*, ΔpXO1, ΔpXO2). Bacterial burden were determined post mortem at various tissues of individual animals.

**Figure 4:**
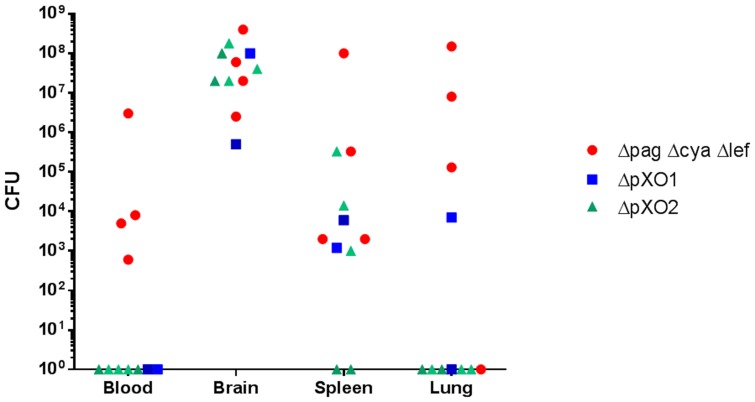
Bacterial dissemination to different organs following IC infection of Rabbits. Rabbits were inoculated IC with capsular bacteria of the different Vollum strain mutants (Δ*pag*Δ*lef*Δ*cya*, ΔpXO1, ΔpXO2). Bacterial burden were determined post mortem at various tissues of individual animals.

The hypothesis that the VollumΔpXO1 lacks the neuro-invasive trait gains surprising support from a comparison of bacterial loads in different organs upon death following IC inoculation with Vollum strains (5×10^5^ CFU). As can be seen in **Fig. 3**, in GP VollumΔpXO1 is restricted to the brain, and is not found in any other organ. On the other hand, VollumΔ*pag*Δ*lef*Δ*cya*, which causes lethal infection following SC inoculation, was found in several tissues following IC injection. Similarly, in the rabbit the neuro-invasive strain VollumΔ*pag*Δ*lef*Δ*cya* was found to localize to several tissues following IC injection, however the noninvasive mutants did show limited leakage from the brain to other tissues, mainly the spleen (**Fig. 4**). Therefore, it seems that there is a correlation between the ability of the mutant to invade the brain from the bloodstream and the ability to “escape” from the brain back into the bloodstream, following IC inoculation; a mutant that can induce a lethal infection by invading the brain from the circulation can also, after IC inoculation, spread into the circulation by "escaping" out of the brain, whereas a "non-invasive" mutant is likewise unable to exit the brain.

Similar to the pattern seen in GP, mutants carrying either pXO1 or pXO2 have a lethal effect in rabbits when injected IC, whereas deletion of both plasmids will abolish any virulence (**Table 1**). Here again the lethal effect of the VollumΔpXO2 mutant is toxin dependent, as deletion of the *pag, lef* and *cya* genes abolishes this effect. Furthermore, the difference in the lethality in rabbits of the Vollum mutants following IC injections and that seen following IV injections (**Table 2**), is exemplified by the encapsulated strain VollumΔpXO1, which is lethal when injected IC, but asymptomatic when inoculated IV. As this discrepancy may indicate that this mutant lacks the ability to induce a fatal brain infection, we can again conclude that neuro-invasiveness is AtxA-dependent, and indeed, all IC virulent mutants carrying the *atxA* will kill the rabbits when injected IV.

**Table 2:** Virulence of Vollum strains in rabbits and GP.

Strain		Vollum wt	Vollum Δ*pag* Δ*lef* Δ*cya*	Vollum ΔpXO1	Vollum ΔpXO2	Vollum Δ*pag* Δ*lef* Δ*cya* Δ*atxA*	Vollum ΔpXO1 *ba2805:: atxA*	VollumΔ*pag* Δ*lef* Δ*cya* ΔpXO2	Vollum ΔpXO1 ΔpXO2
pXO1/pXO2		+/+	+/+	−/+	+/−	+/+	+/−	+/−	−/−
**Rb**	**SC**	V	V (HD)	NV	NV	NV	ND	ND	ND
	**IV**	V	V	NV	V (HD)	NV	V	ND	ND
	**IC**	V	V	V	V	V	ND	NV	NV
**GP**	**SC**	V	V	NV	V (HD)	NV	V	ND	ND
	**IC**	V	V	V	V	ND	ND	NV	NV

V- virulent, HD- high dose, NV- non virulent, ND – not done.

**Figure 5:**
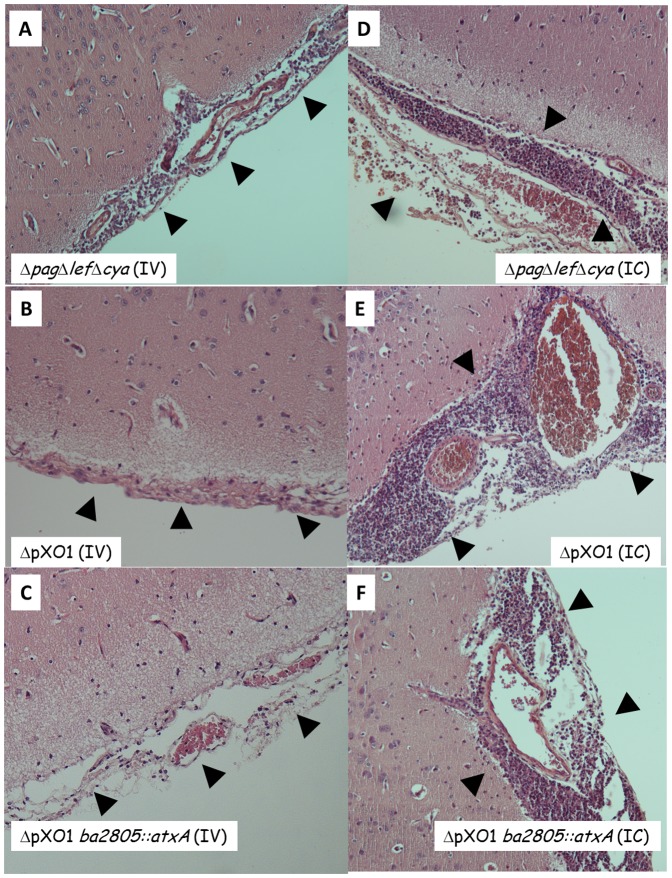
H&E staining of meninges from rabbits that were infected IV or IC with different mutants. Strain genotype and infection route are as indicated. Normal meninges histology (tight membranes without significant immune cell infiltration) was observed in panel B -ΔpOX1- whereas pathological inflammation can be detected in all the rest (A, C-F). Panels A-D magnification of x100, panels E and F magnification of x400.

**Figure 6:**
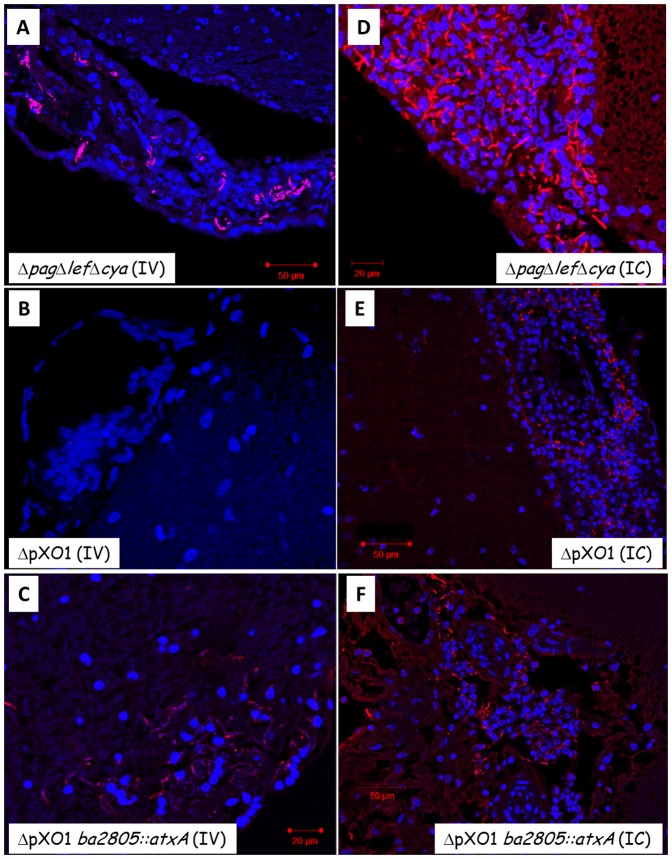
Immuno- histopathology of meninges from rabbits that were infected IV or IC with different mutants. Immuno-staining of tissues (parallel to figure 5) using anti- killed capsular bacteria anti-serum (Atto 594 – red) and cell nuclei (DAPI – blue). Strain genotype and infection route are as indicated. *B. anthracis* were observed and demonstrated by immunodetection with all pathogenic strains (A, C-F), but was not present with the non-pathogenic mutant, ΔpOX1 (panel B). Scale bar is 20–50 µm as indicated.

The findings presented so far can be corroborated by histological examination of rabbits' brains following IV and IC inoculations. As can be seen using H&E staining, VollumΔ*pag*Δ*lef*Δ*cya* shows patterns consistent with acute meningitis (edema and significant infiltration of monocytes and polymorphonuclear cells) following either IV (**Fig 5A**) or IC (**Fig 5D**) inoculation. In contrast, VollumΔpXO1 shows a typical meningitis pattern only following IC inoculation (**Fig 5E**) but not after IV inoculation (**Fig 5B**), where no pathological effects are observed. On the other hand, insertion of the atxA gene into the genome of the non-virulent Vollum ΔpXO1, creating the VollumΔpXO1*ba2805::atxA* strain, resulted in the recovery of the virulent trait and the induction of a lethal brain infection (**Fig 5C, F** **,** **Fig. 6C, F**), without altering the overall wt dissemination to other tissues (data not shown, [3]). Immuno-staining corroborates these findings showing that the virulent strain VollumΔ*pag*Δ*lef*Δ*cya* accumulates in the brain tissue following IV inoculation (**Fig 6A**), whereas the non-virulent ΔpXO1 mutant cannot be detected in the brain at all (**Fig 6B**). Introducing these mutants IC resulted in bacterial accumulation (**Fig 6D-F**). Therefore, we can conclude that the toxin-independent virulent trait of *B. anthracis* relies on its ability to invade the brain and is regulated by AtxA.

## Discussion

The accepted paradigm states that anthrax is both an invasive and toxinogenic disease and that the toxins play a major role in pathogenicity. In previous studies we and others have suggested that the main role of the toxins is during the early stages of the infection, enabling the organism to overcome innate host defenses, whereas the death of the animals relates to bacteremia and organ bacterial burden/damage rather than to systemic toxemia. Reduction of the toxemic load by hyper-immune sera did not save bacteremic animals [12], [13]. Furthermore, the deletion of all three toxin components, *pag, lef* and *cya* genes, results only in partial attenuation, maintaining significant virulence of these mutants. The finding that virulence is maintained after fully deleting the toxin components suggests that *B*. *anthracis* possesses an additional virulence mechanism, which is toxin independent.

This virulent trait was studied in rabbits and GP by a systematic genetic approach using various routes of inoculation: SC, IV and now IC. A schematic summary of the results obtained in these studies is shown in **Table 2**.

The fully virulent Vollum-wt strain will induce the development of a lethal disease in both animal models by any route of inoculation. However, deletion of the plasmids pXO1 and pXO2 will result in complete avirulence. Deletion of pXO1 abolishes the virulence exerted by bacteria inoculated peripherally, either SC or IV, but retains lethality when injected IC, indicating that pXO2 is sufficient to kill the host when this mutant reaches the brain. On the other hand, VollumΔpXO2, carrying pXO1, will kill the host when injected IC, in a toxin-dependent mechanism, as deletion of the toxin-genes will abolish the virulent trait. Thus, it seems that either the capsule or the toxins suffice to ensure killing of the host upon reaching the CNS, indicating that a certain amount of redundancy exists at this stage of the disease between the toxins and the capsule in terms of the protection afforded to the bacteria against the brain defense systems.

The outstanding difference between the two routes of inoculation in both animal models is exhibited by the encapsulated strain VollumΔpXO1, which is lethal when injected IC, but asymptomatic when inoculated SC. These results can be interpreted as if pXO1 mediates brain invasion in the virulent strains. By analyzing the contribution of the entire pXO1 vs. the toxins and/or AtxA to this neuro-invasive trait, we demonstrated that AtxA plays a major role in this ability. VollumΔ*pag*Δ*lef*Δ*cya*, carrying pXO1 but lacking the toxin-encoding genes, although attenuated when compared to the fully virulent Vollum-wt, exhibits significant virulence independent of the route of inoculation. This mutant loses its peripheral virulence when the *atxA* gene is also deleted (VollumΔ*pag*Δ*lef*Δ*cya*Δ*atxA*) [3]. On the other hand, insertion of the *atxA* gene into the genome of the non-virulent VollumΔpXO1 (VollumΔpXO1*ba2805::atxA*) results in the recovery of the virulent trait. Therefore, we can conclude that the *atxA* gene is the only component of pXO1 required for the exhibition of the invasive trait. This conclusion is corroborated by the histological findings shown in **Figures 5** and **6**, demonstrating the localization of the virulent strains VollumΔ*pag*Δ*lef*Δ*cya* and VollumΔpXO1*ba2805::atxA* to the rabbit brain following IV inoculation, whereas the non-virulent strain VollumΔpXO1 could not be detected in the brain.

Bacterial invasion into the brain is not an artifact of the mutants used or the route of inoculation, but rather an intermediate step in the development of the anthrax disease. IN instillation of Vollum-wt spores in rabbits (**Fig. 2**), an accepted model for inhalational anthrax, is followed by the development of bacteremia. The appearance of bacteria in the brain occurs at high levels of bacteremia, quickly reaching bacterial levels that surpass the contribution of bacteremic blood in the brain vasculature. Furthermore, high levels of bacteria in the brain are accompanied by presence of bacteria in the CSF. Monitoring bacterial dissemination following lethal IC inoculation reveals an interesting phenomenon. Whereas the invasive strain, VollumΔ*pag*Δ*cya*Δ*lef*, disseminates to the blood and tissues, the non-invasive strain, VollumΔpXO1, can be detected only (GP) or mainly (rabbit) in the brain. The neuro-invasive mutant can also escape, whereas a non-invasive mutant, lacking the *atxA* gene, is unable to exit the brain. This phenomenon cannot be explained in terms of circulatory survival as both mutants, VollumΔ*pag*Δ*cya*Δ*lef* and VollumΔpXO1 were shown to survive and persist in the bloodstream [3]. The AtxA-dependent brain invasion could be mediated by an active mechanism or rely on the AtxA-dependent protection to the bacteria in the circulation. If there is any correlation between the ability of the bacteria to invade and to exit the brain, these finding would oppose the circulation survival explanation.

Attachment of blood-borne bacteria to brain endothelium (or choroid plexus epithelium) and subsequent invasion into the brain may represent the initial step in penetration or disruption of the BBB. BslA, a putative surface layer immunoreactive protein encoded by pXO1, was studied and shown to mediate adherence of Sterne vegetative bacteria to host cells [15]–[17], including to blood-brain barrier endothelial cells, promoting penetration during the pathogenesis of anthrax meningitis in a bacteremia dependent manner [18]. However, in our hands, deletion of the *bslA* gene in the VollumΔ*pag*Δ*cya*Δ*lef* background had a minor effect on the pathogenicity of the mutant [3] (mainly prolonging the mean time to death). Furthermore, we demonstrated in the encapsulated strains (pXO2^+^) that *atxA* is the only gene in pXO1 required for the exertion of brain invasion.

Elucidation of the mechanism by which AtxA mediates brain penetration may be of major importance, as Anthrax meningitis is the main neurological complication of systemic infection, approaching 100% mortality despite intensive antibiotic therapy [9].

On the other hand, artificially-induced VollumΔ*pag*Δ*cya*Δ*lef* bacteremia in animals could be cured by passive immunization [3], preventing bacterial meningitis. The AtxA dependent virulence mechanism should be further explored to discover the immunogen responsible for this curative effect (the involvement of candidate genes such as anthrolysin (ALO) and inhA [19] has been ruled out – **Table S1**). Therefore we assume that elucidation of the neuroinvasion mechanism may have major implications on future research both on *B. anthracis* pathogenicity and on vaccine development.

## Material and Methods

### Bacterial strains, media and growth conditions

*B. anthracis* strains used in this study are listed in **Table 3**. A detailed description of these strains and the methodology used for their construction was previously published [2]. *B. anthracis* strains were cultivated in Terrific broth [20] at 37°C with vigorous shaking (250 rpm). For the induction of toxins and capsule production, DMEM-10% FBS was used. Sporulation was carried out using G broth, as previously described [21].

**Table 3:** Bacterial strains used in this study.

	Description/characteristics	Source
Strain		
***B. anthracis***		
Vollum	ATCC 14578	IIBR collection
VollumΔ*pag*Δ*cya*Δ*lef*	Complete deletion of the *pag*, *lef* and *cya* genes	[2]
VollumΔ*pag*Δ*cya*Δ*lef*Δ*atxA*	Complete deletion of the *atxA* gene in the VollumΔ*pag*Δ*cya*Δ*lef* mutant	
VollumΔ*pag*Δ*cya*Δ*lef*	Complete deletion of the *bslA* gene in the VollumΔ*pag*Δ*cya*Δ*lef* mutant	
VollumΔpXO1*BA2805*::*atxA*	Genome insertion of the *atxA* gene replacing major parts of the PlyPH (*BA2805*) [22] in the VollumΔpXO1	[3]
VollumΔ*pag*Δ*cya*Δ*lef*ΔpXO2	Curing of the pXO2 plasmid in the VollumΔ*pag*Δ*cya*Δ*lef* mutant	
VollumΔpXO1	Vollum pXO1-, pXO2+	IIBR Collection
VollumΔpXO2	Vollum pXO1+, pXO2-	IIBR Collection
VollumΔpXO1ΔpXO2	Vollum pXO1-, pXO2-	IIBR Collection

### Infection of rabbits and guinea pigs

New Zealand white female rabbits (2.2–2.5 kg) were used to test the virulence of the wild-type and mutant Vollum strains. Spores were germinated by incubation in Terrific broth for 1 hr, and then incubated in DMEM-10% FBS for 2 hr, at 37°C with 10% CO_2_ to induce capsule formation. The capsule was visualized by negative staining with India ink. The encapsulated vegetative bacteria were used to infect rabbits and GP.

Intravenous inoculation in rabbits was performed as described previously [3].

For intracranial (IC) infection; 30 min prior to the procedure, rabbits were injected SC in the area of the surgery with 0.1 ml/kg Calmagin. Rabbits were anesthetized using 2∶1 mix of Xylazine Ketamine (1.5 ml). Prior to the surgery Lidocaine (0.3 ml) was injected intradermal and the animals' head were fixed in a stereotactic apparatus. A 3–4 cm longitudinal incision was made along the sagittal suture of the skull, centered around the bregma (the coronal-suture's transection of the sagittal suture). The underlying connective tissue was removed to expose the skull. A small hole (1 mm diameter) was drilled 5 mm caudally and 5 mm laterally from the bregma using a handheld power drill (Dremel 300, Dremel, Illinois, USA). Encapsulated vegetative bacteria (30 µl) were injected through the drilled hole at a 4-mm depth from the dura surface and the scalp was closed using surgical clips. A remaining sample was plated for total viable counts (CFU.ml^−1^). The animals were observed daily for 14 days or for the indicated period. Upon death, blood samples were plated and DNA was extracted, followed by PCR analysis in order to determine the identity of the strain responsible for the animals' death.

Female Hartley guinea pigs (Charles River Laboratories), weighing 220–250 g were used.

Guinea pigs were immunized subcutaneously with 0.5 ml alum absorbed PA [12] at time 0 and 4 weeks and challenged at week 6 by intranasal instillation of spores.

For IC inoculation, the animals were anesthetized and medicated as described above and the animal's head was fixed to the table. A 3 cm midline incision was made from a point posterior to the eyes line along the sagittal suture, and the skull exposed. A hole was drilled in the skull (similarly to those done in rabbits) 3 mm caudally and 3 mm laterally to the bregma. Encapsulated vegetative bacteria (30 µl) were injected through the drilled hole at a 3-mm depth from the dura surface and the scalp was closed using surgical clips. The remaining suspensions were plated for total viable counts (CFU.ml^−1^). The animals were observed daily for 14 days. Upon death, blood samples were plated and DNA was extracted, followed by PCR analysis in order to verify the genotype of the strain responsible for the animals' death.

The animals were infected intranasally (IN) with spore preparations of either the mutant strains or the parental Vollum strain. Prior to infecting the animals, the spore preparations were heat-shocked (70°C, 20 min) and serially diluted in saline to produce spore suspensions of 10^7^–10^8^ per ml. A spore dose of 0.1 ml (GP) or 1 ml (rabbit) was administered IN to each animal. The remaining spore dose suspensions were plated for total viable counts (CFU.ml^−1^). The animals were observed daily for 14 days or for the indicated period. Upon death, blood samples were plated and DNA was extracted, followed by PCR analysis in order to confirm the identity of the strain responsible for the animals' death.

The animal were maintained under 12 h light 12 h dark regime, room temperature of 22–24°C for the duration of the experiment.

### Determination of bacterial burden in tissues

Spleen, lungs, brain, kidneys and liver were harvested postmortem from rabbits and GP that succumbed to the infection and the organs were placed in a 50 ml tube containing sterile PBS and immediately homogenized. Serial dilutions of the homogenate were plated on agar plates to determine bacterial loads. The total volume of lysate was recorded and CFU counts were corrected to total bacterial burden per organ.

### Tissue processing for histopathology

Brains chosen for histological analysis were harvested postmortem from rabbits and GP that succumbed to the infection, or at designated time point (controls and non-lethal infections). The brains were immediately placed in 50 ml tubes containing ∼30 ml of 3.7% formaldehyde in PBS for fixation. After fixation, brains were cross-sectioned into 4–5 mm thick slices, each placed in a separate histological cassette, and the slices were paraffinized overnight in a Leica APS200 system (Leica Biosystems, Wetzler, Germany). The tissue slices were then embedded in paraffin blocks and consequently slides were prepared by mounting 5 µm thick sections prepared using a rotary microtome (Leica Biosystems, Wetzler, Germany).

### Histopathological staining

Prepared sections were subjected to either H&E staining (using a protocol modified for brain staining - 15 min hematoxylin instead of the usual duration) or immunofluorescence staining for bacteria.

The immunofluorescence protocol included deparaffinizaiton, and then staining with a polyclonal Rb serum against formalin killed vegetative capsular bacteria (produced by our group) as the primary antibody and a commercial donkey-anti-rabbit-Atto-594 antibody as well as DAPI in the secondary staining phase. For negative control we used a staining with only the secondary antibody. Coverslips were mounted with Fluoromount (Sigma).

### Image acquisition

H&E slide images were acquired using a Zeiss Axiokop microscope (Zeiss, Oberkochen, Germany) equipped with a Nikon DS-Ri1 camera controlled by a DS-U3 Digital Sight and the Nis-Elements-Br software suite (Nikon, Tokyo, Japan). Fluorescent images were acquired using a Zeiss LSM 710 confocal microscopy system (Zeiss, Oberkochen, Germany).

### Ethics Statement

This study was carried out in strict accordance with the recommendations of the Guide for the Care and Use of Laboratory Animals of the National Research Council. The protocols were approved by the Committee on the Ethics of Animal Experiments of the Israel Institute for Biological Research (permit numbers GP-11-2012; GP-03-2013; RB-26-2012; RB-27-2012). Animals were euthanized when one of the following symptoms was detected: severe respiratory distress or the loss of righting reflex. Guinea pigs were sacrificed by CO_2_ inhalation and rabbits by the sodium pentabarbitone injection.

## Supporting Information

Table S1 **Mutation that do not affect the toxin independent virulence in the rabbit IV model.**
(DOCX)
