# Supplementary material for: The Central Nervous System as Target of Bacillus anthracis Toxin Independent Virulence in Rabbits and Guinea Pigs
Source: PLoS One. 2014 Nov 6;9(11):e112319. doi: 10.1371/journal.pone.0112319 (PMC4223028; doi:10.1371/journal.pone.0112319)
Supplement: Table S1 — Mutation that do not affect the toxin independent virulence in the rabbit IV model. (DOCX) [file pone.0112319.s001.docx]

**Table S1: mutation that do not affect the toxin independent virulence in the rabbit IV model**

| **Genotype** | **Inoculum**  **(CFU)** | **Dead/**  **infected** | **MTTD**  **(days)** |
| --- | --- | --- | --- |
| Vollum Δ*pag*Δ*cya*Δ*lef* | 5x10^6^ | 4/4 | 1 |
|  |  |  |  |
| Vollum ΔpXO1 | 10^8^ | 0/4 | >14 |
|  |  |  |  |
| Vollum Δ*pag*Δ*cya*Δ*lef*Δ***ALO*** | 10^7^ | 2/2 | 1 |
|  |  |  |  |
| Vollum Δ*pag*Δ*cya*Δ*lef*Δ***inhA1*** | 10^7^ | 2/2 | 1 |
|  |  |  |  |
| Vollum Δ*pag*Δ*cya*Δ*lef*Δ***inhA1*Δ*inhA2*** | 5x10^6^ | 2/2 | 1 |
|  |  |  |  |
| Vollum Δ*pag*Δ*cya*Δ*lef*Δ***sap*Δ*eag*** | 5x10^6^ | 2/2 | 1 |
|  |  |  |  |

Rabbits were inoculated IV with vegetative cells of the specific mutants strains.
